# 2-Methyl-4-oxo-6,7,8,9-tetrahydro­thieno[2′,3′:4,5]pyrimidino­[1,2-*a*]pyridine-3-carboxylic acid

**DOI:** 10.1107/S1600536811007902

**Published:** 2011-03-09

**Authors:** Burkhon Zh. Elmuradov, Khurshed A. Bozorov, Rasul Ya. Okmanov, Bakhodir Tashkhodjaev, Khusnutdin M. Shakhidoyatov

**Affiliations:** a S.Yunusov Institute of the Chemistry of Plant Substances, Academy of Sciences of Uzbekistan, Mirzo Ulugbek St 77, Tashkent 100170, Uzbekistan

## Abstract

There are two independent mol­ecules in the asymmetric unit of the title compound, C_12_H_12_N_2_O_3_S. With the exception of the methyl­ene groups, a mean plane fitted through all non-H atoms of each mol­ecule has an r.m.s. deviation of 0.035 Å for one mol­ecule and 0.120 Å for the second. In one of the independent mol­ecules, the methyl­ene groups was refined using a disorder model with an occupancy ratio of 0.53:0.47 (14). Each molecule features an intra­molecular O—H⋯O hydrogen bond, which generates an *S*(7) ring.

## Related literature

For the synthesis of thieno[2,3-*d*]pyrimidin-4-ones and their derivatives, see: Litvinov (2004 ▶); Elmuradov *et al.* (2010 ▶); Csukonyi *et al.* (1986 ▶). For the physiological activity of thieno[2,3-*d*]pyrimidin-4-ones and their derivatives, see: Lilienkampf *et al.* (2007 ▶). For a related structure, see: Bozorov *et al.* (2010 ▶). For hydrogen-bond motifs, see: Bernstein *et al.* (1995 ▶).
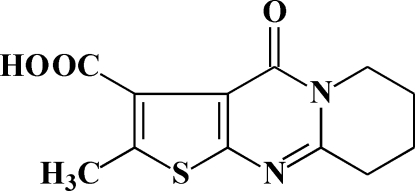


## Experimental

### 

#### Crystal data

C_12_H_12_N_2_O_3_S
*M* *_r_* = 264.30
Monoclinic, 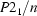

*a* = 7.2550 (15) Å
*b* = 20.506 (4) Å
*c* = 15.824 (3) Å
β = 96.93 (3)°
*V* = 2337.0 (8) Å^3^
*Z* = 8
Cu *K*α radiation
μ = 2.50 mm^−1^
*T* = 296 K
0.60 × 0.40 × 0.15 mm

#### Data collection

Stoe STADI4 diffractometer
Absorption correction: ψ scan (Blessing, 1987 ▶) *T*_min_ = 0.346, *T*_max_ = 0.687
4297 measured reflections
3438 independent reflections
2420 reflections with *I* > 2σ(*I*)
*R*_int_ = 0.047
θ_max_ = 60.0°
3 standard reflections every 60 min  intensity decay: 4.7%

#### Refinement

*R*[*F* ^2^ > 2σ(*F* ^2^)] = 0.063
*wR*(*F* ^2^) = 0.169
*S* = 1.10
3438 reflections
348 parameters
23 restraints
H atoms treated by a mixture of independent and constrained refinement
Δρ_max_ = 0.20 e Å^−3^
Δρ_min_ = −0.28 e Å^−3^

### 

Data collection: *STADI4* (Stoe & Cie, 1997 ▶); cell refinement: *STADI4*; data reduction: *X-RED* (Stoe & Cie, 1997 ▶); program(s) used to solve structure: *SHELXS97* (Sheldrick, 2008 ▶); program(s) used to refine structure: *SHELXL97* (Sheldrick, 2008 ▶); molecular graphics: *SHELXTL* (Sheldrick, 2008 ▶); software used to prepare material for publication: *publCIF* (Westrip, 2010 ▶).

## Supplementary Material

Crystal structure: contains datablocks I, global. DOI: 10.1107/S1600536811007902/nk2082sup1.cif

Structure factors: contains datablocks I. DOI: 10.1107/S1600536811007902/nk2082Isup2.hkl

Additional supplementary materials:  crystallographic information; 3D view; checkCIF report

## Figures and Tables

**Table 1:** Hydrogen-bond geometry (Å, °)

*D*—H⋯*A*	*D*—H	H⋯*A*	*D*⋯*A*	*D*—H⋯*A*
O2—H2*O*⋯O1	0.87 (2)	1.63 (2)	2.501 (5)	177 (7)
O52—H52*O*⋯O51	0.87 (2)	1.71 (3)	2.518 (6)	154 (4)

**Fig. 1.**
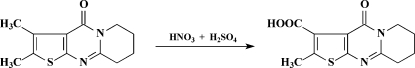
Reaction scheme.

**Fig. 2.**
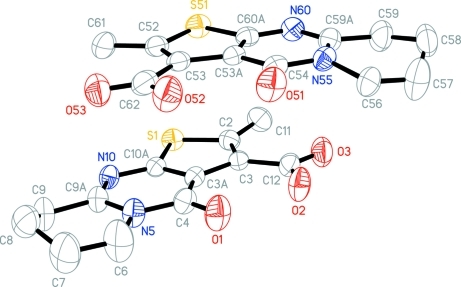
The asymmetric unit of the title compound, with H atoms and the minor disorder component omitted. Displacement ellipsoids are at the 30% probability level.

## supplementary crystallographic information

### Comment

Among heterocyclic compounds. the thieno[2,3-*d*]pyrimidin-4-one family (Litvinov, 2004; Elmuradov *et al.*, 2010; Csukonyi *et al.*, 1986) has been shown to possess various physiological activity (Lilienkampf *et al.*, 2007). The reaction of 2,3-dimethylthieno[2',3':4,5]pyrimidino[1,2-*a*]pyridin-4-one with nitric acid in ratio of reagents - substrate:HNO_3_ - 1:4 in concentrated sulfuric acid leads to the formation of 3-hydroxycarbonyl-2- methylthieno[2',3':4,5]pyrimidino[1,2-*a*]pyridin-4-one (Figure 1). We report here the synthesis and crystal structure.

The asymmetric unit contains two crystallographically unique molecules (Figure 2). With the exception of the methylene group, a mean plane fitted through all non-H atoms of each molecule has an rms deviation of 0.035 for one molecule, and 0.120 for the second. In one of the unique molecules the methylene group was refined using a disorder model with an occupancy ratio of 0.53:0.47 (14). An S(7) intramolecular O—H···O hydrogen bond is observed in each unique molecule (Bernstein *et al.*, 1995).

### Experimental

Into a flask supplied with a mixer was poured 2 ml H_2_SO_4_ and cooled by an ice bath (0.25 h). Then 1 g (4.26 mmole) 2,3-dimethylthieno[2',3':4,5]pyrimidino[1,2-*a*]pyridin-4-one was added in portions, mixed before complete dissolution, nitrating acid consisting of 1.89 g (1.4 ml, d═1.35 g/ml) (17.08 mmole) HNO_3_ and 1.4 ml (d═1.835 g/ml) H_2_SO_4_ were added (0.5 h) drop wise. A reactionary mixture were mixed 1 h at room temperature and left 2 days at room temperature and a mixture is decomposed in cold. The formed yellow crystals were filtered off and washed with water and dried. Yield 1.0 g (89%), m.p. 478–479 K (ethanol). The yellow crystals suitable for X-ray analysis were obtained from absolute methanol at room temperature.

### Refinement

C-bound H atoms were placed geometrically (with C—H distances of 0.97 Å for CH_2_; 0.96 Å for CH_3_; and 0.93 Å for C_ar_) and included in the refinement in a riding motion approximation with *U*_iso_=1.2*U*_eq_(C) [*U*_iso_=1.5*U*_eq_(C) for methyl H atoms]. O-bound H atoms involved in the intermolecular hydrogen bonding were found by difference Fourier synthesis and refined isotropically with a distance restrains of 0.85 (2) Å. [O2—H2O = 0.870 (20) Å, O52—H52O = 0.871 (19) Å].  Atoms C56, C57, C58 and C59 were refined using a disorder model with an occupancy ratio of 0.53:0.47 (14)

### Crystal data

**Table:**

C_12_H_12_N_2_O_3_S	*F*(000) = 1104
*M**_r_* = 264.30	*D*_x_ = 1.502 Mg m^−^^3^
Monoclinic, *P*2_1_/*n*	Cu *K*α radiation, λ = 1.54184 Å
Hall symbol: -P 2yn	Cell parameters from 12 reflections
*a* = 7.2550 (15) Å	θ = 10–20°
*b* = 20.506 (4) Å	µ = 2.50 mm^−^^1^
*c* = 15.824 (3) Å	*T* = 296 K
β = 96.93 (3)°	Prism, yellow
*V* = 2337.0 (8) Å^3^	0.60 × 0.40 × 0.15 mm
*Z* = 8	

### Data collection

**Table:**

Stoe STADI4 diffractometer	2420 reflections with *I* > 2σ(*I*)
Radiation source: fine-focus sealed tube	*R*_int_ = 0.047
graphite	θ_max_ = 60.0°, θ_min_ = 3.5°
Scan width (ω) = 1.56 – 1.80, scan ratio 2θ:ω = 1.00 I(Net) and sigma(I) calculated according to Blessing (1987)	*h* = −8→8
Absorption correction: ψ scan (Blessing, 1987)	*k* = 0→23
*T*_min_ = 0.346, *T*_max_ = 0.687	*l* = 0→17
4297 measured reflections	3 standard reflections every 60 min
3438 independent reflections	intensity decay: 4.7%

### Refinement

**Table:**

Refinement on *F*^2^	Secondary atom site location: difference Fourier map
Least-squares matrix: full	Hydrogen site location: inferred from neighbouring sites
*R*[*F*^2^ > 2σ(*F*^2^)] = 0.063	H atoms treated by a mixture of independent and constrained refinement
*wR*(*F*^2^) = 0.169	*w* = 1/[σ^2^(*F*_o_^2^) + (0.0601*P*)^2^ + 2.3488*P*] where *P* = (*F*_o_^2^ + 2*F*_c_^2^)/3
*S* = 1.10	(Δ/σ)_max_ < 0.001
3438 reflections	Δρ_max_ = 0.20 e Å^−^^3^
348 parameters	Δρ_min_ = −0.28 e Å^−^^3^
23 restraints	Extinction correction: *SHELXL*, Fc^*^=kFc[1+0.001xFc^2^λ^3^/sin(2θ)]^-1/4^
Primary atom site location: structure-invariant direct methods	Extinction coefficient: 0.0018 (3)

### Special details

**Table:**

Experimental. ψ Scan Reflections used µ * R = 0.00 H K L, θ, χ, I_min_/I_max_: -1 -1 0 13.0 84.8 0.563^1^H NMR (400 MHz, CDCl_3_): 15.46 (1*H*, s, OH), 4.06 (2*H*, t, J═6.1 Hz, H-6), 3.01 (2*H*, t, J═6.6 Hz, H-9), 2.76 (3*H*, s, CH_3_-2), 1.93–2.07 (4*H*, m, H-7, 8).
Geometry. All esds (except the esd in the dihedral angle between two l.s. planes) are estimated using the full covariance matrix. The cell esds are taken into account individually in the estimation of esds in distances, angles and torsion angles; correlations between esds in cell parameters are only used when they are defined by crystal symmetry. An approximate (isotropic) treatment of cell esds is used for estimating esds involving l.s. planes.
Refinement. Refinement of F^2^ against ALL reflections. The weighted R-factor wR and goodness of fit S are based on F^2^, conventional R-factors R are based on F, with F set to zero for negative F^2^. The threshold expression of F^2^ > 2sigma(F^2^) is used only for calculating R-factors(gt) etc. and is not relevant to the choice of reflections for refinement. R-factors based on F^2^ are statistically about twice as large as those based on F, and R- factors based on ALL data will be even larger.

### Fractional atomic coordinates and isotropic or equivalent isotropic displacement parameters (Å^2^)

**Table:**

	*x*	*y*	*z*	*U*_iso_*/*U*_eq_	Occ. (<1)
S1	0.04758 (17)	0.79444 (6)	0.42793 (7)	0.0671 (4)	
O1	0.2734 (6)	0.78157 (17)	0.7344 (2)	0.0980 (12)	
O2	0.3136 (6)	0.66799 (17)	0.6820 (3)	0.0995 (13)	
H2O	0.304 (9)	0.7074 (15)	0.701 (4)	0.119*	
O3	0.2595 (6)	0.61070 (18)	0.5666 (3)	0.1034 (13)	
N5	0.1590 (6)	0.87759 (18)	0.6804 (2)	0.0722 (11)	
N10	0.0491 (5)	0.89415 (17)	0.5367 (2)	0.0619 (10)	
C2	0.1269 (6)	0.7205 (2)	0.4683 (3)	0.0651 (12)	
C3	0.1810 (6)	0.7225 (2)	0.5538 (3)	0.0580 (11)	
C3A	0.1573 (5)	0.7869 (2)	0.5876 (2)	0.0538 (10)	
C4	0.2008 (7)	0.8123 (2)	0.6711 (3)	0.0680 (13)	
C6	0.2045 (12)	0.9053 (3)	0.7664 (3)	0.132 (3)	
H6A	0.1358	0.8810	0.8048	0.159*	
H6B	0.3354	0.8977	0.7841	0.159*	
C7	0.1711 (14)	0.9690 (3)	0.7773 (4)	0.146 (3)	
H7A	0.2672	0.9851	0.8202	0.175*	
H7B	0.0546	0.9722	0.8013	0.175*	
C8	0.1608 (11)	1.0132 (3)	0.7067 (4)	0.114 (2)	
H8A	0.2849	1.0221	0.6927	0.136*	
H8B	0.1070	1.0542	0.7223	0.136*	
C9	0.0451 (9)	0.9853 (2)	0.6305 (4)	0.0967 (18)	
H9A	−0.0848	0.9889	0.6388	0.116*	
H9B	0.0642	1.0110	0.5809	0.116*	
C9A	0.0868 (7)	0.9159 (2)	0.6132 (3)	0.0679 (12)	
C10A	0.0859 (6)	0.8302 (2)	0.5262 (2)	0.0533 (10)	
C11	0.1308 (8)	0.6661 (3)	0.4061 (3)	0.0911 (17)	
H11A	0.2570	0.6528	0.4036	0.137*	
H11B	0.0770	0.6804	0.3509	0.137*	
H11C	0.0610	0.6299	0.4239	0.137*	
C12	0.2532 (7)	0.6630 (3)	0.6007 (4)	0.0745 (14)	
S51	0.54855 (18)	0.78491 (7)	0.40855 (7)	0.0753 (4)	
O51	0.7745 (6)	0.74580 (18)	0.71060 (19)	0.0938 (12)	
O52	0.7039 (7)	0.8660 (2)	0.7032 (3)	0.1067 (14)	
H52O	0.756 (9)	0.8281 (18)	0.715 (4)	0.129*	
O53	0.5648 (7)	0.9368 (2)	0.6164 (3)	0.1136 (15)	
N55	0.7534 (5)	0.65997 (19)	0.6203 (2)	0.0672 (10)	
N60	0.6566 (5)	0.66871 (19)	0.4739 (2)	0.0684 (10)	
C52	0.5490 (6)	0.8492 (2)	0.4774 (3)	0.0683 (13)	
C53A	0.6617 (5)	0.7637 (2)	0.5653 (2)	0.0535 (10)	
C53	0.6134 (6)	0.8322 (2)	0.5592 (3)	0.0615 (11)	
C54	0.7323 (6)	0.7256 (2)	0.6369 (3)	0.0642 (12)	
C56	0.792 (9)	0.6213 (13)	0.7004 (13)	0.089 (7)	0.468 (14)
H56A	0.9111	0.6347	0.7298	0.106*	0.468 (14)
H56B	0.6979	0.6312	0.7370	0.106*	0.468 (14)
C57	0.795 (7)	0.5546 (14)	0.6872 (16)	0.138 (8)	0.468 (14)
H57A	0.6742	0.5381	0.6969	0.166*	0.468 (14)
H57B	0.8840	0.5366	0.7319	0.166*	0.468 (14)
C58	0.838 (3)	0.5262 (9)	0.6076 (14)	0.104 (4)	0.468 (14)
H58A	0.8013	0.4807	0.6053	0.125*	0.468 (14)
H58B	0.9703	0.5285	0.6050	0.125*	0.468 (14)
C59	0.737 (8)	0.5619 (7)	0.5322 (15)	0.107 (3)	0.468 (14)
H59A	0.8012	0.5534	0.4832	0.129*	0.468 (14)
H59B	0.6134	0.5432	0.5203	0.129*	0.468 (14)
C56'	0.832 (7)	0.6170 (11)	0.6917 (12)	0.089 (7)	0.532 (14)
H56C	0.9576	0.6318	0.7108	0.106*	0.532 (14)
H56D	0.7597	0.6230	0.7387	0.106*	0.532 (14)
C57'	0.840 (6)	0.5505 (13)	0.6745 (15)	0.138 (8)	0.532 (14)
H57C	0.8124	0.5275	0.7251	0.166*	0.532 (14)
H57D	0.9668	0.5401	0.6665	0.166*	0.532 (14)
C58'	0.719 (3)	0.5234 (7)	0.6019 (12)	0.104 (4)	0.532 (14)
H58C	0.5906	0.5249	0.6130	0.125*	0.532 (14)
H58D	0.7519	0.4782	0.5932	0.125*	0.532 (14)
C59'	0.744 (7)	0.5631 (6)	0.5233 (13)	0.107 (3)	0.532 (14)
H59C	0.8676	0.5563	0.5077	0.129*	0.532 (14)
H59D	0.6549	0.5490	0.4762	0.129*	0.532 (14)
C59A	0.7158 (7)	0.6339 (2)	0.5402 (3)	0.0706 (13)	
C60A	0.6315 (6)	0.7326 (2)	0.4885 (3)	0.0587 (11)	
C61	0.4852 (9)	0.9139 (3)	0.4415 (4)	0.0991 (19)	
H61A	0.3715	0.9259	0.4628	0.149*	
H61B	0.4649	0.9112	0.3805	0.149*	
H61C	0.5783	0.9462	0.4582	0.149*	
C62	0.6225 (8)	0.8821 (3)	0.6276 (4)	0.0836 (15)	

### Atomic displacement parameters (Å^2^)

**Table:**

	*U*^11^	*U*^22^	*U*^33^	*U*^12^	*U*^13^	*U*^23^
S1	0.0690 (8)	0.0763 (8)	0.0541 (6)	0.0001 (6)	0.0000 (5)	0.0014 (6)
O1	0.138 (3)	0.083 (2)	0.064 (2)	0.010 (2)	−0.025 (2)	0.0097 (18)
O2	0.132 (3)	0.069 (2)	0.091 (3)	0.012 (2)	−0.014 (2)	0.020 (2)
O3	0.135 (4)	0.062 (2)	0.114 (3)	0.012 (2)	0.018 (2)	0.004 (2)
N5	0.094 (3)	0.060 (2)	0.058 (2)	0.001 (2)	−0.009 (2)	−0.0030 (18)
N10	0.065 (2)	0.056 (2)	0.062 (2)	0.0013 (18)	−0.0050 (18)	0.0081 (18)
C2	0.062 (3)	0.065 (3)	0.068 (3)	−0.006 (2)	0.011 (2)	−0.007 (2)
C3	0.051 (3)	0.056 (3)	0.069 (3)	−0.001 (2)	0.012 (2)	0.004 (2)
C3A	0.050 (2)	0.056 (3)	0.054 (2)	−0.004 (2)	0.0020 (18)	0.006 (2)
C4	0.076 (3)	0.066 (3)	0.058 (3)	−0.004 (2)	−0.004 (2)	0.006 (2)
C6	0.230 (9)	0.094 (5)	0.064 (4)	0.010 (5)	−0.022 (4)	−0.022 (3)
C7	0.252 (11)	0.097 (5)	0.089 (5)	−0.004 (6)	0.016 (5)	−0.026 (4)
C8	0.164 (7)	0.083 (4)	0.097 (4)	−0.021 (4)	0.030 (4)	−0.023 (4)
C9	0.115 (5)	0.058 (3)	0.112 (4)	0.006 (3)	−0.008 (4)	−0.001 (3)
C9A	0.072 (3)	0.055 (3)	0.074 (3)	0.002 (2)	−0.003 (2)	0.002 (2)
C10A	0.048 (2)	0.056 (3)	0.055 (2)	−0.003 (2)	0.0022 (18)	0.003 (2)
C11	0.100 (4)	0.091 (4)	0.083 (4)	0.001 (3)	0.010 (3)	−0.018 (3)
C12	0.071 (3)	0.061 (3)	0.093 (4)	−0.004 (3)	0.014 (3)	0.011 (3)
S51	0.0716 (8)	0.0943 (10)	0.0577 (7)	0.0027 (7)	−0.0013 (6)	0.0137 (6)
O51	0.132 (3)	0.094 (3)	0.0509 (19)	−0.001 (2)	−0.0072 (19)	0.0014 (18)
O52	0.162 (4)	0.082 (3)	0.076 (3)	−0.003 (3)	0.016 (3)	−0.009 (2)
O53	0.153 (4)	0.072 (3)	0.121 (3)	0.013 (3)	0.041 (3)	−0.002 (2)
N55	0.072 (3)	0.067 (3)	0.062 (2)	−0.003 (2)	0.0049 (19)	0.0116 (19)
N60	0.075 (3)	0.068 (3)	0.062 (2)	−0.006 (2)	0.0056 (19)	−0.008 (2)
C52	0.058 (3)	0.078 (3)	0.070 (3)	0.002 (2)	0.014 (2)	0.014 (2)
C53A	0.047 (2)	0.062 (3)	0.052 (2)	−0.006 (2)	0.0081 (19)	0.005 (2)
C53	0.055 (3)	0.066 (3)	0.066 (3)	−0.007 (2)	0.019 (2)	0.000 (2)
C54	0.063 (3)	0.073 (3)	0.056 (3)	−0.007 (2)	0.008 (2)	0.004 (2)
C56	0.105 (19)	0.086 (5)	0.074 (5)	0.014 (6)	0.009 (7)	0.031 (4)
C57	0.202 (19)	0.090 (6)	0.116 (9)	0.012 (8)	−0.011 (10)	0.030 (6)
C58	0.107 (10)	0.064 (4)	0.138 (7)	0.019 (9)	0.005 (12)	0.012 (5)
C59	0.147 (7)	0.059 (4)	0.120 (6)	0.002 (4)	0.030 (6)	−0.002 (4)
C56'	0.105 (19)	0.086 (5)	0.074 (5)	0.014 (6)	0.009 (7)	0.031 (4)
C57'	0.202 (19)	0.090 (6)	0.116 (9)	0.012 (8)	−0.011 (10)	0.030 (6)
C58'	0.107 (10)	0.064 (4)	0.138 (7)	0.019 (9)	0.005 (12)	0.012 (5)
C59'	0.147 (7)	0.059 (4)	0.120 (6)	0.002 (4)	0.030 (6)	−0.002 (4)
C59A	0.073 (3)	0.064 (3)	0.075 (3)	−0.004 (2)	0.009 (3)	−0.001 (3)
C60A	0.047 (2)	0.075 (3)	0.054 (2)	−0.003 (2)	0.0043 (19)	0.004 (2)
C61	0.107 (5)	0.085 (4)	0.108 (4)	0.012 (3)	0.024 (3)	0.039 (3)
C62	0.090 (4)	0.080 (4)	0.085 (4)	−0.013 (3)	0.027 (3)	0.005 (3)

### Geometric parameters (Å, °)

**Table:**

S1—C10A	1.712 (4)	N55—C54	1.382 (6)
S1—C2	1.717 (5)	N55—C56	1.492 (13)
O1—C4	1.245 (5)	N55—C56'	1.492 (12)
O2—C12	1.313 (6)	N60—C59A	1.299 (6)
O2—H2O	0.87 (2)	N60—C60A	1.346 (6)
O3—C12	1.204 (6)	C52—C53	1.367 (6)
N5—C9A	1.374 (5)	C52—C61	1.495 (6)
N5—C4	1.384 (6)	C53A—C60A	1.368 (5)
N5—C6	1.474 (6)	C53A—C54	1.420 (6)
N10—C9A	1.288 (5)	C53A—C53	1.447 (6)
N10—C10A	1.352 (5)	C53—C62	1.485 (7)
C2—C3	1.363 (6)	C54—O51	1.241 (5)
C2—C11	1.491 (6)	C56—C57	1.385 (13)
C3—C3A	1.444 (6)	C56—H56A	0.9700
C3—C12	1.489 (6)	C56—H56B	0.9700
C3A—C10A	1.370 (5)	C57—C58	1.45 (2)
C3A—C4	1.421 (6)	C57—H57A	0.9700
C4—O1	1.245 (5)	C57—H57B	0.9700
C6—C7	1.342 (7)	C58—C59	1.512 (18)
C6—H6A	0.9700	C58—H58A	0.9700
C6—H6B	0.9700	C58—H58B	0.9700
C7—C8	1.435 (8)	C59—C59A	1.490 (13)
C7—H7A	0.9700	C59—H59A	0.9700
C7—H7B	0.9700	C59—H59B	0.9700
C8—C9	1.498 (7)	C56'—C57'	1.392 (12)
C8—H8A	0.9700	C56'—H56C	0.9700
C8—H8B	0.9700	C56'—H56D	0.9700
C9—C9A	1.487 (6)	C57'—C58'	1.47 (2)
C9—H9A	0.9700	C57'—H57C	0.9700
C9—H9B	0.9700	C57'—H57D	0.9700
C11—H11A	0.9600	C58'—C59'	1.516 (18)
C11—H11B	0.9600	C58'—H58C	0.9700
C11—H11C	0.9600	C58'—H58D	0.9700
S51—C52	1.709 (5)	C59'—C59A	1.494 (12)
S51—C60A	1.711 (4)	C59'—H59C	0.9700
O51—C54	1.241 (5)	C59'—H59D	0.9700
O52—C62	1.312 (7)	C61—H61A	0.9600
O52—H52O	0.871 (19)	C61—H61B	0.9600
O53—C62	1.202 (6)	C61—H61C	0.9600
N55—C59A	1.372 (6)		
			
C10A—S1—C2	91.7 (2)	C52—C53—C53A	111.3 (4)
C12—O2—H2O	113 (4)	C52—C53—C62	119.6 (5)
C9A—N5—C4	122.6 (4)	C53A—C53—C62	129.1 (4)
C9A—N5—C6	121.0 (4)	O51—C54—N55	118.9 (4)
C4—N5—C6	116.4 (4)	O51—C54—N55	118.9 (4)
C9A—N10—C10A	115.4 (4)	O51—C54—C53A	126.1 (5)
C3—C2—C11	130.6 (5)	O51—C54—C53A	126.1 (5)
C3—C2—S1	112.8 (3)	N55—C54—C53A	115.0 (4)
C11—C2—S1	116.6 (4)	C57—C56—N55	114 (2)
C2—C3—C3A	111.1 (4)	C57—C56—H56A	108.8
C2—C3—C12	120.8 (4)	N55—C56—H56A	108.8
C3A—C3—C12	128.0 (4)	C57—C56—H56B	108.8
C10A—C3A—C4	116.4 (4)	N55—C56—H56B	108.8
C10A—C3A—C3	112.5 (4)	H56A—C56—H56B	107.7
C4—C3A—C3	131.0 (4)	C56—C57—C58	122 (3)
O1—C4—N5	118.8 (4)	C56—C57—H57A	106.8
O1—C4—N5	118.8 (4)	C58—C57—H57A	106.8
O1—C4—C3A	126.0 (4)	C56—C57—H57B	106.8
O1—C4—C3A	126.0 (4)	C58—C57—H57B	106.8
N5—C4—C3A	115.2 (4)	H57A—C57—H57B	106.6
C7—C6—N5	118.0 (6)	C57—C58—C59	110.9 (19)
C7—C6—H6A	107.8	C57—C58—H58A	109.5
N5—C6—H6A	107.8	C59—C58—H58A	109.5
C7—C6—H6B	107.8	C57—C58—H58B	109.5
N5—C6—H6B	107.8	C59—C58—H58B	109.5
H6A—C6—H6B	107.1	H58A—C58—H58B	108.0
C6—C7—C8	120.6 (6)	C59A—C59—C58	117.1 (17)
C6—C7—H7A	107.2	C59A—C59—H59A	108.0
C8—C7—H7A	107.2	C58—C59—H59A	108.0
C6—C7—H7B	107.2	C59A—C59—H59B	108.0
C8—C7—H7B	107.2	C58—C59—H59B	108.0
H7A—C7—H7B	106.8	H59A—C59—H59B	107.3
C7—C8—C9	110.9 (5)	C57'—C56'—N55	116.8 (18)
C7—C8—H8A	109.5	C57'—C56'—H56C	108.1
C9—C8—H8A	109.5	N55—C56'—H56C	108.1
C7—C8—H8B	109.5	C57'—C56'—H56D	108.1
C9—C8—H8B	109.5	N55—C56'—H56D	108.1
H8A—C8—H8B	108.1	H56C—C56'—H56D	107.3
C9A—C9—C8	114.1 (5)	C56'—C57'—C58'	119 (2)
C9A—C9—H9A	108.7	C56'—C57'—H57C	107.4
C8—C9—H9A	108.7	C58'—C57'—H57C	107.4
C9A—C9—H9B	108.7	C56'—C57'—H57D	107.4
C8—C9—H9B	108.7	C58'—C57'—H57D	107.5
H9A—C9—H9B	107.6	H57C—C57'—H57D	107.0
N10—C9A—N5	123.2 (4)	C57'—C58'—C59'	108.7 (19)
N10—C9A—C9	118.6 (4)	C57'—C58'—H58C	110.0
N5—C9A—C9	118.2 (4)	C59'—C58'—H58C	110.0
N10—C10A—C3A	127.2 (4)	C57'—C58'—H58D	110.0
N10—C10A—S1	121.0 (3)	C59'—C58'—H58D	110.0
C3A—C10A—S1	111.8 (3)	H58C—C58'—H58D	108.3
C2—C11—H11A	109.5	C59A—C59'—C58'	110.1 (15)
C2—C11—H11B	109.5	C59A—C59'—H59C	109.6
H11A—C11—H11B	109.5	C58'—C59'—H59C	109.6
C2—C11—H11C	109.5	C59A—C59'—H59D	109.6
H11A—C11—H11C	109.5	C58'—C59'—H59D	109.6
H11B—C11—H11C	109.5	H59C—C59'—H59D	108.2
O3—C12—O2	118.8 (5)	N60—C59A—N55	122.8 (4)
O3—C12—C3	122.5 (5)	N60—C59A—C59	120.2 (10)
O2—C12—C3	118.7 (5)	N55—C59A—C59	117.0 (10)
C52—S51—C60A	92.2 (2)	N60—C59A—C59'	115.4 (9)
C62—O52—H52O	124 (3)	N55—C59A—C59'	121.9 (9)
C59A—N55—C54	122.7 (4)	N60—C60A—C53A	126.6 (4)
C59A—N55—C56	124.9 (12)	N60—C60A—S51	121.8 (3)
C54—N55—C56	111.7 (12)	C53A—C60A—S51	111.6 (3)
C59A—N55—C56'	119.0 (10)	C52—C61—H61A	109.5
C54—N55—C56'	118.2 (10)	C52—C61—H61B	109.5
C59A—N60—C60A	115.8 (4)	H61A—C61—H61B	109.5
C53—C52—C61	129.8 (5)	C52—C61—H61C	109.5
C53—C52—S51	112.4 (4)	H61A—C61—H61C	109.5
C61—C52—S51	117.8 (4)	H61B—C61—H61C	109.5
C60A—C53A—C54	117.1 (4)	O53—C62—O52	118.6 (6)
C60A—C53A—C53	112.4 (4)	O53—C62—C53	123.5 (6)
C54—C53A—C53	130.5 (4)	O52—C62—C53	117.9 (5)
			
C10A—S1—C2—C3	0.1 (4)	C59A—N55—C54—O51	−177.8 (4)
C10A—S1—C2—C11	−178.4 (4)	C56—N55—C54—O51	11 (3)
C11—C2—C3—C3A	178.0 (5)	C56'—N55—C54—O51	−2(2)
S1—C2—C3—C3A	−0.2 (5)	C59A—N55—C54—O51	−177.8 (4)
C11—C2—C3—C12	−2.1 (8)	C56—N55—C54—O51	11 (3)
S1—C2—C3—C12	179.7 (3)	C56'—N55—C54—O51	−2(2)
C2—C3—C3A—C10A	0.3 (5)	C59A—N55—C54—C53A	2.4 (6)
C12—C3—C3A—C10A	−179.6 (4)	C56—N55—C54—C53A	−169 (3)
C2—C3—C3A—C4	−177.3 (4)	C56'—N55—C54—C53A	178 (2)
C12—C3—C3A—C4	2.8 (8)	C60A—C53A—C54—O51	177.1 (5)
C9A—N5—C4—O1	177.0 (5)	C53—C53A—C54—O51	−3.2 (8)
C6—N5—C4—O1	−0.2 (8)	C60A—C53A—C54—O51	177.1 (5)
C9A—N5—C4—O1	177.0 (5)	C53—C53A—C54—O51	−3.2 (8)
C6—N5—C4—O1	−0.2 (8)	C60A—C53A—C54—N55	−3.1 (6)
C9A—N5—C4—C3A	−2.2 (7)	C53—C53A—C54—N55	176.6 (4)
C6—N5—C4—C3A	−179.5 (5)	C59A—N55—C56—C57	3(6)
C10A—C3A—C4—O1	−176.9 (5)	C54—N55—C56—C57	174 (4)
C3—C3A—C4—O1	0.6 (8)	C56'—N55—C56—C57	−65 (10)
C10A—C3A—C4—O1	−176.9 (5)	N55—C56—C57—C58	25 (7)
C3—C3A—C4—O1	0.6 (8)	C56—C57—C58—C59	−44 (6)
C10A—C3A—C4—N5	2.2 (6)	C57—C58—C59—C59A	37 (5)
C3—C3A—C4—N5	179.7 (4)	C59A—N55—C56'—C57'	−9(5)
C9A—N5—C6—C7	0.7 (11)	C54—N55—C56'—C57'	176 (3)
C4—N5—C6—C7	178.0 (8)	C56—N55—C56'—C57'	111 (15)
N5—C6—C7—C8	−23.3 (14)	N55—C56'—C57'—C58'	−20 (6)
C6—C7—C8—C9	45.4 (12)	C56'—C57'—C58'—C59'	52 (4)
C7—C8—C9—C9A	−45.3 (8)	C57'—C58'—C59'—C59A	−53 (4)
C10A—N10—C9A—N5	−0.1 (7)	C60A—N60—C59A—N55	−0.8 (7)
C10A—N10—C9A—C9	−178.2 (5)	C60A—N60—C59A—C59	176 (3)
C4—N5—C9A—N10	1.2 (8)	C60A—N60—C59A—C59'	−180 (2)
C6—N5—C9A—N10	178.3 (6)	C54—N55—C59A—N60	−0.4 (7)
C4—N5—C9A—C9	179.4 (5)	C56—N55—C59A—N60	170 (3)
C6—N5—C9A—C9	−3.6 (8)	C56'—N55—C59A—N60	−176 (2)
C8—C9—C9A—N10	−155.3 (5)	C54—N55—C59A—C59	−177 (3)
C8—C9—C9A—N5	26.4 (8)	C56—N55—C59A—C59	−7(4)
C9A—N10—C10A—C3A	0.2 (7)	C56'—N55—C59A—C59	7(4)
C9A—N10—C10A—S1	−178.8 (3)	C54—N55—C59A—C59'	179 (2)
C4—C3A—C10A—N10	−1.3 (7)	C56—N55—C59A—C59'	−11 (4)
C3—C3A—C10A—N10	−179.3 (4)	C56'—N55—C59A—C59'	3(3)
C4—C3A—C10A—S1	177.7 (3)	C58—C59—C59A—N60	169 (2)
C3—C3A—C10A—S1	−0.2 (5)	C58—C59—C59A—N55	−14 (5)
C2—S1—C10A—N10	179.2 (4)	C58—C59—C59A—C59'	131 (38)
C2—S1—C10A—C3A	0.1 (3)	C58'—C59'—C59A—N60	−152.3 (19)
C2—C3—C12—O3	−3.2 (8)	C58'—C59'—C59A—N55	29 (4)
C3A—C3—C12—O3	176.6 (5)	C58'—C59'—C59A—C59	−9(33)
C2—C3—C12—O2	176.1 (5)	C59A—N60—C60A—C53A	−0.1 (7)
C3A—C3—C12—O2	−4.0 (7)	C59A—N60—C60A—S51	−178.3 (4)
C60A—S51—C52—C53	1.0 (4)	C54—C53A—C60A—N60	2.2 (7)
C60A—S51—C52—C61	179.9 (4)	C53—C53A—C60A—N60	−177.5 (4)
C61—C52—C53—C53A	−179.5 (5)	C54—C53A—C60A—S51	−179.5 (3)
S51—C52—C53—C53A	−0.7 (5)	C53—C53A—C60A—S51	0.8 (5)
C61—C52—C53—C62	1.5 (8)	C52—S51—C60A—N60	177.4 (4)
S51—C52—C53—C62	−179.7 (4)	C52—S51—C60A—C53A	−1.0 (3)
C60A—C53A—C53—C52	0.0 (5)	C52—C53—C62—O53	4.9 (8)
C54—C53A—C53—C52	−179.7 (4)	C53A—C53—C62—O53	−173.9 (5)
C60A—C53A—C53—C62	178.8 (5)	C52—C53—C62—O52	−172.6 (5)
C54—C53A—C53—C62	−0.8 (8)	C53A—C53—C62—O52	8.6 (8)

### Hydrogen-bond geometry (Å, °)

**Table:**

*D*—H···*A*	*D*—H	H···*A*	*D*···*A*	*D*—H···*A*
O2—H2O···O1	0.87 (2)	1.63 (2)	2.501 (5)	177 (7)
O52—H52O···O51	0.87 (2)	1.71 (3)	2.518 (6)	154 (4)

## References

[bb1] Bernstein, J., Davis, R. E., Shimoni, L. & Chang, N.-L. (1995). *Angew. Chem. Int. Ed. Engl.* **34**, 1555–1573.

[bb2] Blessing, R. H. (1987). *Crystallogr. Rev.* **1**, 3–58.

[bb3] Bozorov, K. A., Elmuradov, B. Z., Okmanov, R. Y., Tashkhodjaev, B. & Shakhidoyatov, K. M. (2010). *Acta Cryst.* E**66**, o552–o553. 10.1107/S1600536810004101 PMC2983712 21580322

[bb4] Csukonyi, K., Lazar, J., Bernath, G., Hermecz, I. & Meszaros, Z. (1986). *Monatsh. Chem.* **117**, 1295–1303.

[bb5] Elmuradov, B. Zh., Bozorov, Kh. A. & Shakhidoyatov, Kh. M. (2010). *Khim. Get. Soedin.* pp. 1717–1724.

[bb6] Lilienkampf, A., Heikkinen, S., Mutikainen, I. & Wähäla, K. (2007). *Synthesis*, pp. 2699–2705.

[bb7] Litvinov, V. P. (2004). *Izv. Akad. Nauk. Ser. Khim.* **3**, 463–490.

[bb8] Sheldrick, G. M. (2008). *Acta Cryst.* A**64**, 112–122. 10.1107/S0108767307043930 18156677

[bb9] Stoe & Cie (1997). *STADI4* and *X-RED* Stoe & Cie, Darmstadt, Germany.

[bb10] Westrip, S. P. (2010). *J. Appl. Cryst.* **43**, 920–925.

